# Comparative Study of the Structural and Functional Properties of Membrane-Isolated and Isoelectric pH Precipitated Green Lentil Seed Protein Isolates

**DOI:** 10.3390/membranes11090694

**Published:** 2021-09-08

**Authors:** Etinosa C. Osemwota, Adeola M. Alashi, Rotimi E. Aluko

**Affiliations:** 1 Department of Food and Human Nutritional Sciences, University of Manitoba, Winnipeg, MB R3T 2N2, Canada; osemwote@myumanitoba.ca (E.C.O.); oyinolaa@gmail.com (A.M.A.); 2 The Richardson Centre for Functional Foods and Nutraceuticals, University of Manitoba, Winnipeg, MB R3T 2N2, Canada

**Keywords:** lentil protein isolate, legume proteins, isoelectric pH precipitation, membrane ultrafiltration, functional properties, physicochemical characterization, enzyme-assisted extraction, differential scanning calorimetry, scanning electron microscopy

## Abstract

The demand for isolated seed proteins continues to increase but functionality in food systems can be greatly dependent on the extraction method. In this work, we report the physicochemical and functional properties of lentil seed proteins isolated using various protocols. Lentil flour was defatted followed by protein extraction using isoelectric pH precipitation (ISO) as well as NaOH (MEM_NaOH) and NaCl (MEM_NaCl) extractions coupled with membrane ultrafiltration. The MEM_NaCl had significantly (*p* < 0.05) higher protein content (90.28%) than the ISO (86.13%) and MEM_NaOH (82.55%). At pH 3–5, the ISO was less soluble (2.26–11.84%) when compared to the MEM_NaOH (25.74–27.22%) and MEM_NaCl (27.78–40.98%). However, the ISO had higher yield and protein digestibility (48.45% and 89.82%) than MEM_NaOH (35.05% and 77.87%) and MEM_NaCl (13.35% and 77.61%), respectively. Near-UV circular dichroism spectra showed that the MEM_NaOH had loose tertiary conformation at pH 3, 5, 7 and 9 while ISO and MEM_NaCl had more compact structures at pH 7 and 9. The three protein isolates formed better emulsions (lower oil droplet sizes) at pH 7 and 9 when compared to pH 3 and 5. In contrast, foaming capacity was better at pH 5 than pH 3, 7, and 9.

## 1. Introduction

Interest in plant proteins has significantly grown over the past decade [1,2]. This is partly because good proportions of amino acids in plant proteins make them viable alternatives to animal protein sources which might be needed due to various dietary restrictions including related allergenicity, halal consideration, vegetarianism, and so on [3,4]. Plant proteins from various sources including peas, beans, chickpeas, and lentils, have been considered for their versatility, high protein content, low cost, and good nutritional profile [5,6]. Lentils are among the most cultivated legumes in the world, with Canada being the second largest producer and world’s largest lentil exporter [7,8]. Like other legumes, they are rich in more than half of the total amino acids—including arginine, aspartic acid, glutamic acids, and leucine—while they are limiting in some other essential amino acids like threonine, tryptophan, methionine, phenylalanine, and histidine [9]. However, despite the high protein component of legumes, their quality as ingredients to be used in food formulation is dependent on functional properties and behavior during processing, storage, and consumption of food products [10]. As a result, recent research efforts have been geared towards the production of protein concentrates or isolates with good solubility, emulsion, foaming, gelation, and other functional properties, which also contribute to the sensory qualities and consumer acceptability of food products [11,12].

Various techniques involving wet or dry extraction are used to isolate and concentrate proteins obtained from legumes. However, isolation of pulse proteins is easier using wet processes because of their high solubility in both acidic and alkaline conditions [13]. The pH-shift is one of the most commonly used methods for isolating proteins, which involves alkaline solubilisation followed by adjustment to the isoelectric pH to precipitate most of the polypeptides [14]. However, this method has denaturing effects on proteins and negatively impacts their functional properties. For example, Alonso-Miravalles and others [15] investigated lentil proteins isolated using membrane filtration and isoelectric precipitation method. They concluded that the isolated protein obtained from membrane filtration generally had better functional properties, as expected, since membrane filtration concentrates proteins and removes non-protein materials without the harsh extraction and potentially denaturing conditions used during isoelectric precipitation. Nevertheless, the protein isolate obtained from isoelectric precipitation had a significantly higher protein content in comparison. Results obtained from this study clearly indicates the effect of the isolation method on the protein composition as well as the functional properties of the resulting protein isolate or concentrate. In addition, external factors such as pH, ionic strength, temperature, and the presence of enzymes could also alter the structural conformation and functionality of the isolated protein [16].

To the best of our knowledge, there is scant information on the membrane-isolated proteins of lentil seed, an emerging pulse crop with strong potential as a food ingredient. Thus, the present study would provide information on the physicochemical and functional properties of isolated lentil proteins obtained using various extraction techniques and conditions coupled with membrane processes. The information would be useful in effectively identifying potential applications of these lentil proteins as ingredients in the food industry. Therefore, the objectives of this study were to: (a) isolate lentil proteins using enzyme-assisted digestion and NaCl extraction coupled with membrane processing and compare their properties with those of traditional isoelectric pH-precipitated proteins; and (b) determine relationships between the physicochemical and functional properties of the isolated green lentil seed proteins.

## 2. Materials and Methods

### 2.1. Raw Materials and Sample Preparation

Dehulled green lentil seeds were purchased from a local grocery store (Winnipeg, MB, Canada) and stored at −20 °C. The whole seeds were ground into flour with a Coffee Grinder PC2770, then extracted with acetone (1:10, *w*/*v*, flour:acetone ratio) for 2 h to remove lipids and phytochemicals responsible for coloring. The mixture was then filtered through a cheesecloth (grade 90, 40 × 36 thread count), and dried overnight in a fume hood to remove the acetone. The defatting process was repeated twice, and the dried flour was milled again to obtain a fine flour (DEF). The defatted flour was packed in plastic bags, sealed, and stored at −20 °C. Unstained protein markers (10–200 kDa) for sodium dodecyl sulfate polyacrylamide gel electrophoresis (SDS-PAGE) were purchased from Fisher Scientific (Oakville, ON, Canada). Double-distilled water (DDW) produced from Millipore milli-Q™ water purification system (Millipore Corp., Milford, MA, USA) was used during this research, and analytical grade chemicals used were purchased from either Fisher Scientific or Sigma Aldrich (St. Louis, MO, USA).

#### 2.1.1. Isoelectric Precipitation of Lentil Seed Proteins

The acetone-extracted flour was mixed with DDW at a 5:100 (*w*/*v*) ratio and adjusted to pH 10 using 1 M NaOH to solubilize the proteins. The mixture was continuously stirred for 1 h followed by centrifugation at 5600× *g* for 30 min. The supernatant was collected, filtered with cheesecloth (grade 90, 40 × 36 thread count), adjusted to pH 4.5 with 1 M HCl, stirred for 30 min, and then centrifuged. The resulting precipitate was washed with water to remove contaminating non-protein materials and centrifuged again to obtain the final precipitate, which was mixed with DDW and adjusted to pH 7.0 before freeze-drying as the isoelectric isolate (ISO).

#### 2.1.2. Preparation of Lentil Seed Protein Isolates Using the Membrane Isolation Method

The acetone-extracted flour was mixed with DDW at a 5:100 (*w*/*v*) ratio and adjusted to pH 10 using 1 M NaOH. The mixture was continuously stirred for 1 h, after which it was centrifuged at 5600× *g* for 30 min. The supernatant was collected, filtered with cheesecloth (grade 90, 40 × 36 thread count), adjusted to pH 5.5 with 1 M HCl, then digested with 1% cellulase + 1% α-amylase at 50 °C for 1 h. After digestion, the solution was adjusted to pH 7.0, then cooled down to room temperature as the alkaline extract. The NaCl extraction on the other hand, involved a 5:100 (*w*/*v*) ratio mixture of lentil flour dispersed in 0.1 M NaCl solution, which was also mixed for 1 h and similarly centrifuged to obtain a supernatant, that was labeled the NaCl extract. The alkaline and NaCl extracts were then separately filtered using a 5-kDa ultrafiltration membrane and diluted periodically with DDW until the permeate was clear. Once a clear permeate was obtained, the retentate was collected and then freeze-dried to obtain the membrane isolated alkaline-soluble (MEM_NaOH) or salt-soluble (MEM_NaCl) proteins.

### 2.2. Physicochemical and Functional Properties of Isolated Lentil Proteins

#### 2.2.1. Proximate Composition Analysis

Relevant Association of Official Analytical Chemists’ methods were used to analyze the moisture, dry matter, crude protein, and ash contents of the lentil protein isolates [17]. Crude fibre and fat contents were determined using the methods outlined by the American Oil Chemists’ Society [18]. 

#### 2.2.2. Amino Acid Composition

Amino acid profile of the isolated lentil proteins was analyzed using an HPLC system with a pico-tag column after being digested with 6 M HCl for 24 h [19]. The sulphur containing amino acids (cysteine and methionine), as well as the tryptophan were then determined after performic acid oxidation and alkaline hydrolysis, respectively [20,21]. 

#### 2.2.3. Gel Electrophoresis

Reducing (with mercaptoethanol), non-reducing (without mercaptoethanol), SDS-PAGE of the lentil proteins were carried out using the method outlined by Aluko and McIntosh [22] with minor modifications. Solutions containing Tris-HCl buffer, SDS, and bromophenol blue with (reduced) or without (non-reduced) β-mercaptoethanol, were used in the dissolution of each isolated protein to give a final concentration of 1% (*w*/*v*). All prepared mixtures were separately heated at 100 °C for 10 min, cooled to room temperature, then centrifuged for 10 min at 10,000× *g*. A 1 µL aliquot of the supernatant obtained from each sample was separately loaded onto PhastGel^®^ 8–25% gradient gels and electrophoresis performed with a Phastsystem Separation and Development unit according to the manufacturer’s instructions (Cytiva, Montréal, PQ, Canada). Gels were stained with Coomassie brilliant blue followed by de-staining in water:methanol:acetic acid solution (60:30:10), and finally preserved using a 10% (*v*/*v*) glycerol solution.

#### 2.2.4. Intrinsic Fluorescence

The Intrinsic fluorescence measurements were carried out according to the procedure outlined by Ajibola and others [23]. Stock solutions were prepared by dispersing 10 mg/mL of each protein sample (based on protein weight) in 0.1 M acetate (pH 3 and 5), phosphate (pH 7), and Tris-HCl (pH 9) buffers, followed by centrifuging and collecting the supernatant. The supernatants were diluted to 0.002% (*w*/*v*) using the appropriate buffers and the emission spectra was recorded at 25 °C in a spectrofluorometer (Jasco FP-6300, Tokyo, Japan) equipped with a 1 cm path length quartz microcuvette (150 µL capacity). Protein samples were excited at 280 nm (tyrosine and tryptophan) with emissions recorded from 300 to 500 nm; emissions of the buffer blanks were subtracted from those of the respective samples to obtain the fluorescence spectra. Fmax is the maximum fluorescence intensity (FI) obtained during the wavelength scan, while λmax is the wavelength at Fmax.

#### 2.2.5. Surface Hydrophobicity

Surface hydrophobicity of protein isolates was determined as described by Karaca and others [24], using 1-anilino-8-naphthalenesulfonate (ANS) as the probe. In this method, the hydrophobic groups in the protein are determined due to fluorescent nature of ANS when bound to hydrophobic sites on the protein’s surface. The stock solution (10 mg/mL) of each protein isolate was prepared by mixing sample dispersions (based on protein weight) in a 0.1 M sodium phosphate buffer phosphate buffer (pH 7.0) for 1 h, followed by centrifugation 10,000× *g* for 10 min. The collected supernatants were each diluted to final concentrations of 50, 100, 150, 200, and 250 µL/mL. For each protein concentration, a 20 µL aliquot of 0.8 M ANS solution prepared in 0.1 M sodium phosphate buffer phosphate buffer (pH 7.0) was added. Thereafter, the FI of each mixture was measured at excitation and emission wavelengths of 390 nm and 470 nm, respectively, using the Jasco FP-6300 spectrofluorometer. FI values for the mixtures without ANS were subtracted from those of respective protein solutions containing ANS. The slope (S_0_) of the plot of FI versus protein concentration was calculated by linear regression analysis and used as an index of the protein surface hydrophobicity.

#### 2.2.6. Protein Secondary and Tertiary Structure Measurements by Circular Dichroism (CD)

The far- and near-UV spectra providing information about secondary and tertiary structures of the proteins, respectively were obtained following a previously described method [25]. The protein isolates were solubilized in 0.1 M acetate (pH 3 and 5), phosphate (pH 7), and Tris-HCl (pH 9) buffers, then centrifuged at 10,000× *g* for 30 min. The supernatants were then diluted to 2 and 4 mg/mL concentrations for far- and near-UV spectra measurements, respectively. The far-UV spectra were measured at 190–240 nm using a cuvette with a 0.05 cm path length, while 250–320 nm range was used for near-UV spectra in a cuvette of 0.1 cm path length. All the CD spectra were obtained as the average of three consecutive scans with automatic subtraction of respective buffer spectra. The far-UV data were deconvoluted into secondary structure fractions using the SELCON3 algorithm [26,27] located on the DichroWeb website (http://dichroweb.cryst.bbk.ac.uk/html/home.shtml, accessed on 14 August 2021).

#### 2.2.7. Differential Scanning Calorimetry

Calorimetric measurements were taken using the TA Q100-DSC thermal analyzer (TA Instruments, New Castle, DE). Each protein isolate (100 mg) was accurately weighed into an aluminum pan, hermetically sealed, then heated from 40 °C to 140 °C at a rate of 10 °C/min. A sealed empty pan was used as a reference and all experiments were conducted in duplicates. Onset temperature (T_o_), denaturation temperature (T_d_), and enthalpy of denaturation (∆*H*) were computed using the instrument’s software (Universal Analysis 2000, Version 4.5).

#### 2.2.8. Scanning Electron Microscopy (SEM)

Gels were formed using the least gelling concentration obtained for each sample according to the method of Malomo and others [14] and stored at −20 °C. A thin layer of each of the gels was deposited on a double-sided adhesive carbon tape mounted on an aluminum stub and then coated with a thin gold layer with the help of gold sputter. Micrographs of the samples were observed with a high-resolution Quanta™ FEG 650 Schottky field scanning electron microscope (Hillsboro, OR, USA) at an accelerating potential of 10 kV.

#### 2.2.9. Protein Solubility

Solubility of the protein isolates was determined using the protocol earlier described by Malomo and others [14] with some modifications. In summary, 10 mg of each sample was dispersed in 5 mL of 0.1 M acetate (pH 3 and 5), phosphate (pH 7), or Tris buffers (pH 9) on protein weight basis. The resulting mixture was vortexed thoroughly, hydrated for 1 h, then centrifuged at 5600× *g* for 30 min. The protein content of each supernatant was then determined using the modified Lowry method [28]. The total protein content of the samples was also determined with the same method after dissolving the samples in 0.1 M NaOH solution. The protein solubility (PS) was calculated as

PS (%) = (Protein content of sample at certain pH/Total protein content of sample) × 100(1)

#### 2.2.10. Heat Coagulability (HC)

HC was determined according to the method outlined by Malomo and others [14] which was slightly modified as follows. Protein solutions (10 mg/mL) were prepared in 0.1 M acetate (pH 3 and 5), phosphate (pH 7), and Tris-HCl (pH 9) buffers. The mixtures were heated in a boiling water bath (100 °C) for 15 min, cooled to room temperature, and centrifuged at 5600× *g* for 30 min. The protein contents of the supernatants were determined using the modified Lowry method [28]. HC was obtained by calculating the percentage difference between the supernatant protein content and that of the sample.

#### 2.2.11. Emulsion Formation and Stability

Emulsions were prepared by adding 1 mL of pure canola oil to centrifuge tubes containing aqueous dispersions of the isolated proteins in 0.1 M acetate (pH 3 and 5), phosphate (pH 7), and Tris-HCl (pH 9) buffers. The sample/oil mixtures containing varying protein concentrations (10, 15, or 20 mg/mL) were homogenized at 20,000 rpm for 1 min, using the 12 mm non-foaming shaft on a Polytron PT 3100 homogenizer (Kinematica AG, Lucerne, Switzerland). The oil droplet size (*d*_3,2_) of the emulsions was determined in a Mastersizer 2000 (Malvern Instruments Ltd., Malvern, UK.) with distilled water as dispersant [29]. Using a transfer pipet, emulsions were carefully removed into about 100 mL of constantly sheared, ultrapure water contained in the Hydro 2000S wet sample dispersion component, until the sufficient level of obscuration was achieved. After taking triplicate readings, the mean values were computed as an indicator of their emulsion capacity. Emulsion stability was determined by repeating the measurements to determine the oil droplet size (*d*_3,2_) 30 min after emulsion formation; the original value was expressed as a percentage ratio of the 30 min value. 

#### 2.2.12. Foam Formation and Stability

Foaming properties for each isolated protein were determined according to the method of Chao and others [29] with slight modifications. Protein solutions of various concentrations (10, 15, and 20 mg/mL) were prepared with 0.1 M acetate, phosphate, or Tris-HCl buffer solutions, vortexed thoroughly and left to hydrate for 30 min. Samples were homogenized at 20,000 rpm for 1 min using a 20 mm foaming shaft on the Polytron PT 3100 homogenizer (Kinematica AG, Lucerne, Switzerland). The foam was formed in a 50 mL graduated centrifuge tube to determine the volume of foam formed (foaming capacity). The volume of foam remaining after 30 min at room temperature was expressed as a percent value of original foam volume to obtain foam stability.

Foam Capacity (FC) = (volume after homogenization − volume before homogenization/volume before homogenization) × 100(2)

#### 2.2.13. Water and Oil Holding Capacity

The water and oil holding capacity was determined using the method of Malomo and others [14] with some modifications. Aqueous 40 mg/mL solutions of protein isolates were prepared in pre-weighed 15 mL centrifuge tubes containing phosphate buffers. To determine oil holding capacity, similar sample concentrations were prepared using pure canola oil instead of buffers. The sample dispersion (water or oil) was vortexed thoroughly, then allowed to stand for 30 min at room temperature. The mixtures were centrifuged at 5600× *g* for 30 min and the supernatant containing the excess water or oil was drained for 15 min, after which the centrifuge tubes were reweighed to determine the amount of oil or water retained per gram of protein. 

#### 2.2.14. Least Gelation Concentration (LGC)

The LGC of the isolated proteins was determined using the method of Malomo and others [14]. A series of aqueous protein solutions was prepared in 0.1 M phosphate buffer of concentrations between 2% and 20% (*w*/*v*, based on protein weight). The mixtures were placed into test tubes, vortexed for 5 min, then heated in a water bath at 95 °C for 1 h. The tubes were immediately cooled to room temperature and stored at 4 °C for 14 h. LGC was determined as the minimum protein concentration at which the gel did not slip when the tube was inverted.

#### 2.2.15. In Vitro Protein Digestibility

The in vitro digestibility of the isolated proteins was analysed using the protocols previously outlined by Hsu et al. [30] with some modifications. The protein samples were suspended in an aqueous solution containing DDW and adjusted to pH 8 with 0.1 M NaOH while stirring at 37 °C. A 3 mL aliquot of an enzyme solution (containing 1.6 mg trypsin, 3.1 mg chymotrypsin, and 1.3 mg peptidase/mL) was taken from the enzymatic solution maintained in an ice bath and added to 30 mL of each protein suspension. The drop in pH of the mixture was recorded every 30 s over a 10 min period using a pH meter and the analysis was repeated to obtain duplicate results. The per cent protein digestibility of each protein sample was calculated using the regression equation of Hsu et al. [30] as

% Protein digestibility (Y) = 210.46 − 18.10Xf

where Xf is the final pH value of each sample after a 10 min digestion

#### 2.2.16. Statistical Analysis

Except where indicated, all data were reported as mean ± standard deviation from triplicate determinations. Statistical analysis including one-way ANOVA and the Duncan’s multiple-range tests (*p* < 0.05) were performed using IBM SPSS version 26.

## 3. Results

### 3.1. Proximate Composition

The proximate composition of DEF, ISO, MEM_NaOH, and MEM_NaCl are shown in Table 1. MEM_NaCl had a significantly higher crude protein content when compared to ISO and MEM_NaOH. Fat content was generally low for the defatted flour and protein isolates (<2%), while the carbohydrate content varied significantly with MEM_NaCl having the lowest and DEF having the highest.

### 3.2. Amino Acid Composition

The amino acid composition data of the lentil seed protein isolates were similar with very few notable differences (Table 2). The most abundant amino acids present were glutamic acid (15–17%) and aspartic acid (12%), which included glutamine and asparagine, respectively.

### 3.3. Molecular Weight (MW) Analysis

**Figure 1:**
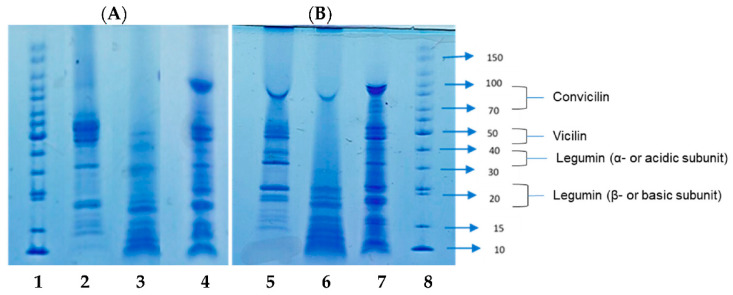
SDS-PAGE of isoelectric pH precipitated isolate (ISO, lanes 4 and 7), and membrane isolates using alkaline (MEM_NaOH, lanes 3 and 6) and salt (MEM_NaCl, lanes 2 and 5) solution extracts under reducing (**A**) and non-reducing (**B**) conditions. Lanes 1 and 8 = standard proteins.

The non-reduced and reduced SDS-PAGE of the lentil seed protein isolates are presented in Figure 1. Under reduced condition, the ISO profile had eight main polypeptides (90, 65, 52, 30, 25, 23, 19, and 15 kDa) with 90 kDa having the highest band intensity. The other isolates under the same condition had different protein profiles, emphasizing the effect of extraction method on their molecular weight distribution. MEM_NaOH had nine polypeptide bands (70, 47, 40, 30, 25, 23, 19, and 15 kDa), while MEM_NaCl had five (57, 30, 23, 19, and 15 kDa) with the highest proportion being 70 kDa and 57 kDa, respectively. The vicilin:legumin ratio ranged from 0.80 to 1.11, with MEM_NaOH having the lowest (Table 3). The vicilins were extracted mostly by NaCl while legumins were mostly solubilized by NaOH. However, the membrane isolates lacked the convicilin band in the presence of mercaptoethanol (Figure 1A) but were present under non-reducing condition (Figure 1B).

### 3.4. Intrinsic Fluorescence

**Figure 2:**
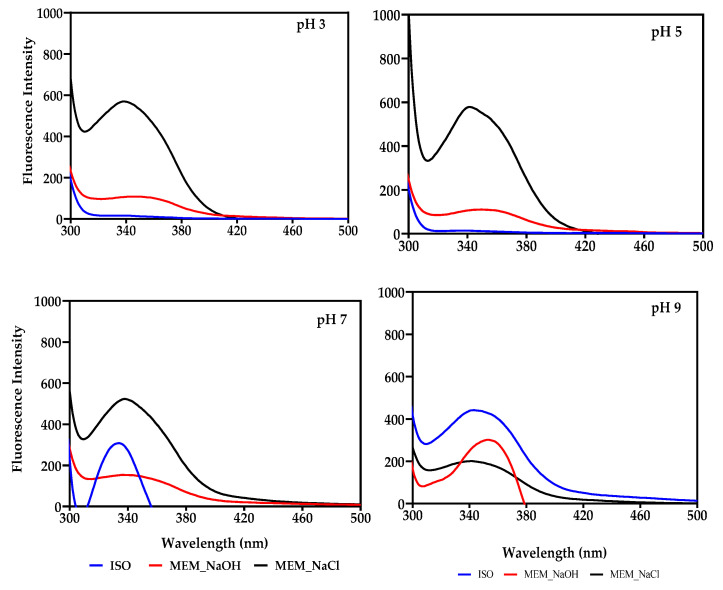
Intrinsic fluorescence intensity at different pH values of isoelectric pH precipitated isolate (ISO), and membrane isolated proteins from alkaline (MEM_NaOH) and salt (MEM_NaCl) solution extracts.

Tryptophan (Trp) and tyrosine (Tyr) residues of the lentil seed protein isolates were excited at a wavelength of 280 nm and showed a single fluorescence peak (Figure 2). The maximum FI occurred at less than 350 nm for all samples. An exception was MEM_NaOH at pH 9, which had a maximum FI at 353 nm, showing about 10 nm red shift compared to ISO and MEM-NaCl at the same pH.

### 3.5. Circular Dichroism (CD)

The effect of pH on secondary structure conformations of the protein isolates was investigated using far-UV CD as shown in Table 3. Generally, more transitions were observed in all samples when shifting from acidic to alkaline mediums suggesting less compact protein structures at pH 3 and 5. The helix structure was the least while unordered was the most fraction in all the protein isolates. There was a general trend of increasing α-helix but decreasing β-sheet conformations when moving from acidic to alkaline pH ranges, except for the MEM_NaCl isolate. However, the three protein isolates showed increasing unordered structure from pH 3 to pH 9.

**Figure 3:**
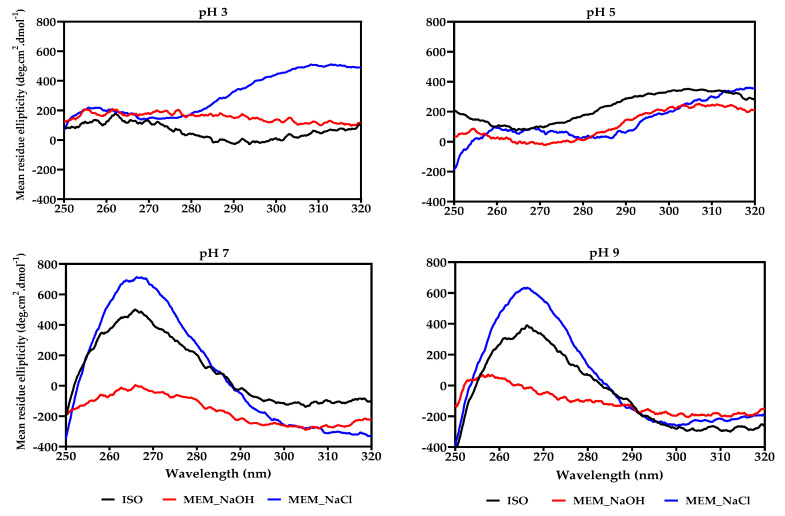
Near_UV circular dichroism spectra at different pH values of isoelectric pH precipitated isolate (ISO), and membrane isolated proteins from alkaline (MEM_NaOH) and salt (MEM_NaCl) solution extracts.

Similar to what was observed in their secondary structure conformations, the near-UV spectra for ISO and MEM_NaOH (Figure 3) were similar but relatively different from that of MEM_NaCl (except pH 9). Nevertheless, change in pH resulted in variations in the tertiary structures of all protein samples. The MEM_NaCl showed a more compact structure than ISO and MEM_NaOH, except at pH 5 where all had similar conformation.

### 3.6. Scanning Electron Microscopy (SEM)

**Figure 4:**
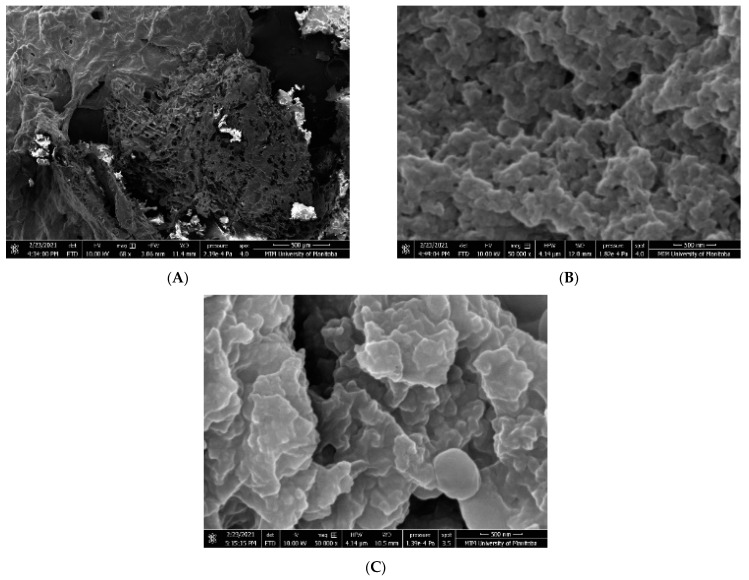
Scanning electron micrographs at 500 µm of: (**A**) isoelectric pH precipitated isolate (ISO); (**B**) membrane isolated proteins from alkaline solution extract (MEM_NaOH); (**C**) membrane isolated proteins from salt solution extract (MEM_NaCl).

Microstructures of the lentil seed protein gels determined using SEM at 500 µm are presented in Figure 4. The SEM depict clumped structures for all lentil protein isolates, especially MEM_NaOH and MEM_NaCl which indicate molecular aggregation of the polypeptides. In addition, MEM_NaOH and MEM_NaCl appear to have a more uniform particle size distribution compared to ISO. Generally, the appearance of smooth but shrivelled surfaces was common with ISO, when compared to MEM_NaOH, and MEM_NaCl.

### 3.7. Differential Scanning Calorimetry (DSC)

Thermal properties of the isolated lentil proteins were analysed using DSC and the thermal transitions are presented in Table 4. The peak denaturation temperature was similar for the protein isolates, but the calculated thermal enthalpy was significantly higher for MEM_NaCl.

### 3.8. Protein Digestibiltiy and Yield

In vitro protein digestibility (IVPD) and yield values of the isolated lentil proteins are presented in Table 5. The protein digestibility of ISO can be seen to be significantly higher than those of the membrane isolates (*p* < 0.05), while that of MEM_NaOH and MEM_NaCl were identical. Similarly, ISO had a significantly higher protein yield than MEM_NaOH and MEM_NaCl.

### 3.9. Surface Hydrophobicity (S_o_)

As shown in Table 5, the S_o_ of MEM_NaCl was higher than that of ISO and MEM_NaOH, implying that there were more exposed hydrophobic clusters in MEM_NaCl and ISO than in MEM_NaOH.

### 3.10. Least Gelation Concentration (LGC), Water (WHC), and Oil Holding Capacity (OHC)

The gelling ability of the lentil protein isolates shown in Table 5 indicates no significant difference between the LGC for ISO, MEM_NaOH, and MEM_NaCl. However, MEM_NaOH had a significantly higher WHC than the other isolated lentil proteins. Conversely, the OHC of ISO was significantly higher when compared to those of MEM_NaOH and MEM_NaCl.

### 3.11. Solubility as a Function of pH

**Figure 5:**
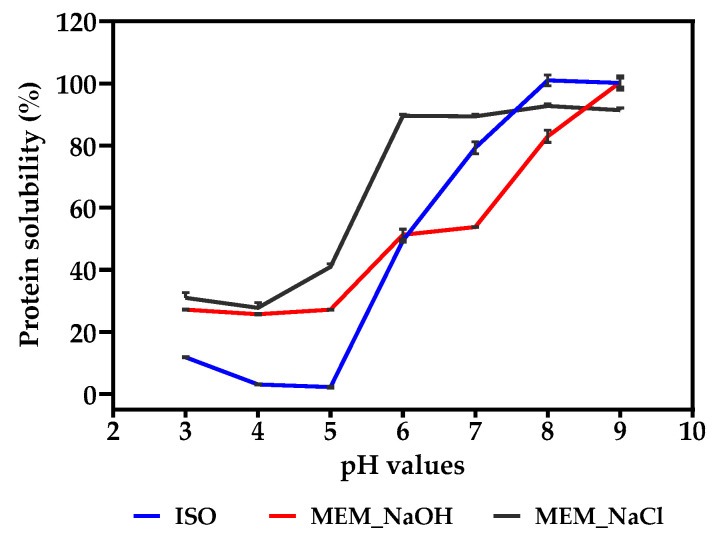
Percentage solubility at pH 3.0–9.0 of isoelectric pH precipitated isolate (ISO), and membrane isolates using alkaline (MEM_NaOH) and salt (MEM_NaCl) solution extracts.

The solubility of the lentil protein isolates was investigated at pH 3 to 9 as presented in Figure 5. Their solubility profiles indicate the highest solubility for ISO and MEM_NaOH at pH 9 (100% and 100%, respectively), with the highest being pH 8 for MEM_NaCl (92%). However, at acidic pH values, the membrane isolated proteins had higher solubility than the isoelectric pH precipitated protein.

### 3.12. Heat Coagulability (HC)

**Figure 6:**
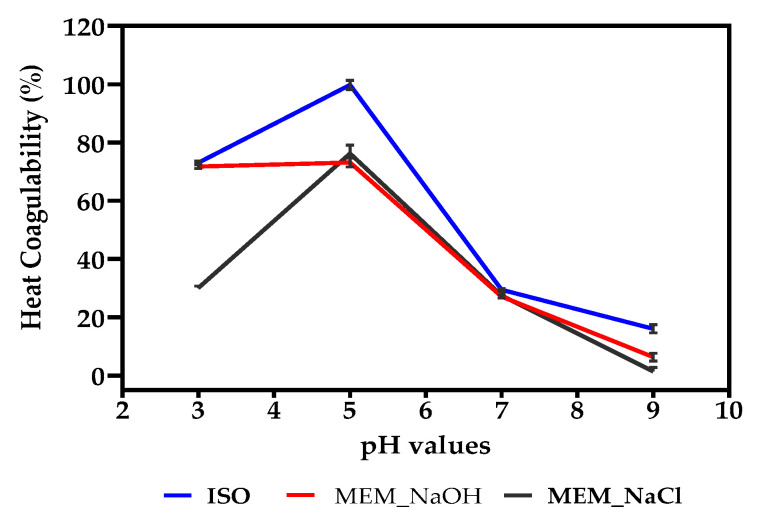
Percentage heat coagulability at pH 3.0–9.0 of isoelectric pH precipitated isolate (ISO), and membrane isolates using alkaline (MEM_NaOH) and salt (MEM_NaCl) solution extracts.

Percent HC was determined as a loss in protein solubility after heating to about 100 °C for 15 min and the results are presented in Figure 6. The protein isolates were most susceptible to heat-induced coagulation at pH 3–6 when compared to pH 7–9. Generally, HC of the membrane isolated proteins was similar but less than that of the isoelectric pH precipitated protein isolate.

### 3.13. Emulsion Formation and Stability

**Figure 7:**
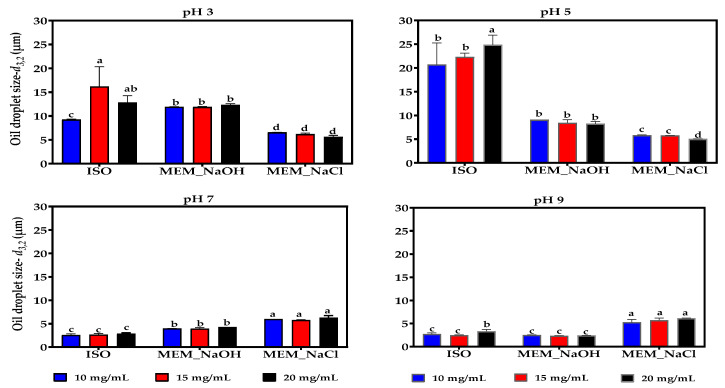
Effect of pH and protein concentration on the oil droplet sizes (*d*_3,2_) of emulsions formed by isoelectric pH precipitated isolate (ISO) and membrane isolated proteins from alkaline (MEM_NaOH) and salt (MEM_NaCl) solution extracts. At each pH, bars with different letters have significantly different (*p* < 0.05) mean values.

**Figure 8:**
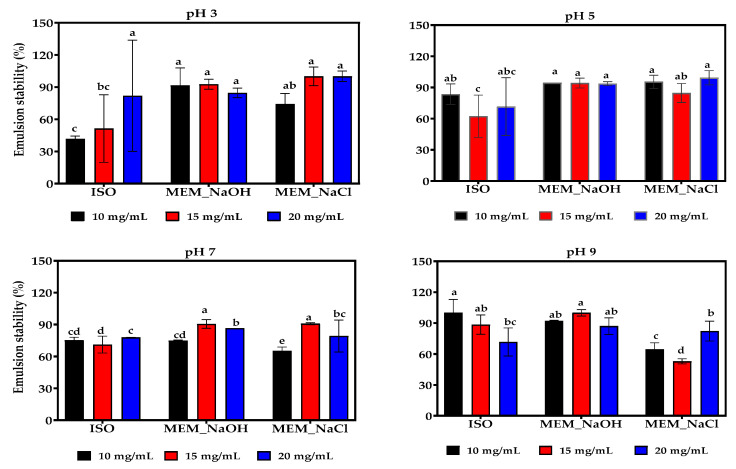
Effect of pH and protein concentration on the stability of emulsions produced with isoelectric pH precipitated isolate (ISO) and membrane isolated proteins from alkaline (MEM_NaOH) and salt (MEM_NaCl) solution extracts. At each pH, bars with different letters have significantly different (*p* < 0.05) mean values.

Changes in the droplet size and stability of the emulsion formed using the isolated lentil proteins were measured at 10 mg/mL, 15 mg/mL, and 20 mg/mL, for pH 3, 5, 7, and 9 (Figure 7 and Figure 8). At acidic pH values and the three protein concentrations, the MEM_NaCl produced emulsions with smaller oil droplet sizes than MEM_NaOH and ISO. However, at pH 5 and the three protein concentrations, the MEM_NaOH emulsions had smaller oil droplet sizes than ISO. In contrast, at pH 7 and 9, the MEM-NaCl produced emulsions with bigger oil droplet sizes than the MEM_NaOH and ISO at the three protein concentrations. Overall, pH had significant effects on emulsion oil droplet size while protein concentration had no measurable effect. The ISO emulsions were most unstable at pH 3 while MEM_NaCl emulsions were least stable at pH 9 when 10 and 15 mg/mL protein concentrations were used for emulsion preparation. For each protein sample, there was no significant effect of protein concentration on emulsion stability.

### 3.14. Foaming Capacity (FC) and Foam Stability (FS)

**Figure 9:**
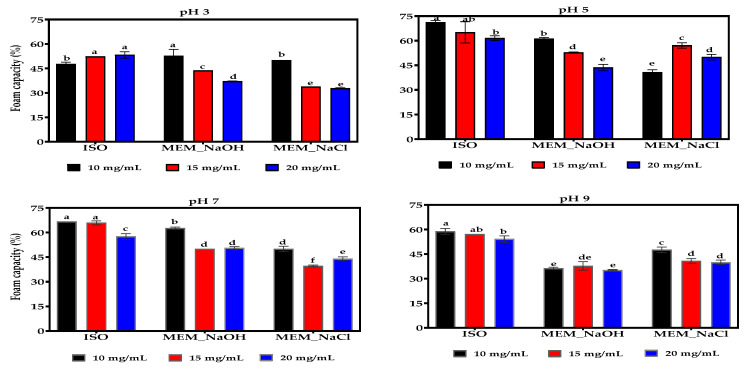
Effect of pH and protein concentration on the foaming capacity of isoelectric pH precipitated isolate (ISO), and membrane isolated proteins from alkaline (MEM_NaOH) and salt (MEM_NaCl) solution extracts. At each pH, bars with different letters have significantly different (*p* < 0.05) mean values.

**Figure 10:**
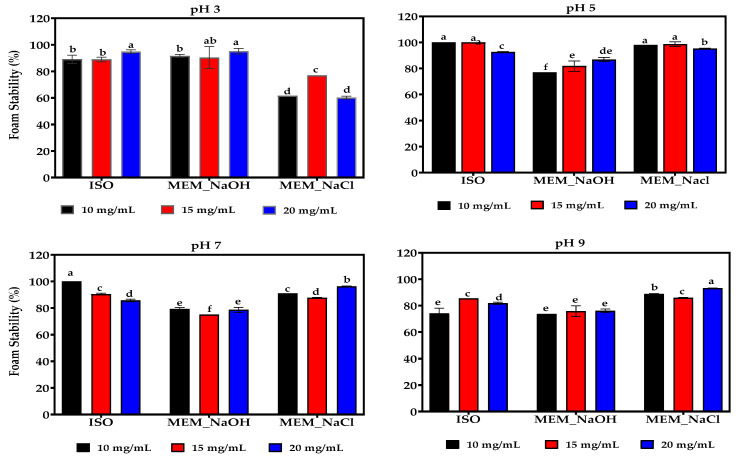
Effect of pH and protein concentration on the foaming stability of isoelectric pH precipitated isolate (ISO), and membrane isolated proteins from alkaline (MEM_NaOH) and salt (MEM_NaCl) solution extracts. At each pH, bars with different letters have significantly different (*p* < 0.05) mean values.

The FC of ISO was significantly higher (*p* < 0.05) than those of MEM_NaOH and MEM_NaCl proteins at most pH values and concentrations (Figure 9). MEM_NaOH had the least FC at pH 9 when compared to the other isolates at the three protein concentrations. Generally, FC decreased with increase in protein concentration except for ISO at pH 3 and MEM_NaCl at pH 5. At pH 3 and the three protein concentrations, the MEM_NaCl had the least FS while MEM_NaOH produced foams with lower stability at pH 5 and 7 when compared to the other two isolates (Figure 10). Unlike the pH, there was no observed effect of protein concentration on FS.

## 4. Discussion

Protein contents of the lentil isolates in Table 1 are comparable to the 85.10–93.7% values that have been reported in scientific literature [15,31,32]. The crude protein content of the defatted lentil flour was the lowest when compared to those of the isolates and is similar to the protein contents of red (29.37%) and green (26.59–27.70%) lentil flours as previously reported [33,34]. The significantly higher amount of crude protein in the isolates compared to the defatted flour emphasises the effectiveness and importance of the various isolation processes used for protein extraction. A negative correlation of the carbohydrate content with the amount of crude proteins present is apparent, with an increasing trend of non-fibre carbohydrates corresponding to a reduced crude protein content. The negative impact of a higher amount of carbohydrates or other non-protein constituents like ash, or fat being co-extracted with proteins is also apparent in the results that have been reported for faba bean, lentil, and soy protein isolates [11]. These proportional differences in the composition of the various isolates play a significant role in predicting some of the measured physicochemical and functional properties.

In general, the amino acid composition data (Table 2) show levels of glutamic + glutamine (13–17%) and aspartic + asparagine (9–11%) that are similar to values previously reported for lentil, pea, and Bambara proteins [35,36,37]. The human body tissues store glutamine and asparagine obtained from foods and this is used by the intestines as the preferable energy supply during the body’s metabolic processes [38], hence the high levels are good for maintaining optimal human health. The next most abundant amino acids are arginine and leucine, as is common with legumes [35,36]. Arginine is a conditionally essential amino acid that generates nitric oxide, which promotes normal endothelial functions, insulin secretion, and pancreatic beta cell protection. Arginine has also been known to have a positive impact on type II diabetes Mellitus by intensifying energy expenditure, improving glucose homeostasis, maintaining lean body mass, and lowering blood lipids as well as blood pressure. Dietary requirement is the amount and quality of amino acids needed to adequately fuel metabolic processes and maintain appropriate body composition and growth. Therefore, the amino acid profile of the protein is pertinent to its perceived nutritive value. All lentil protein isolates were limiting in methionine when compared with the standard amino acid requirements [9]. With the exception of methionine, all other essential amino acids—including tryptophan, threonine, cysteine, valine, isoleucine, histidine, and lysine—were found to be higher than the FAO/WHO requirements for adults. This makes lentil proteins nutritionally superior, especially when paired with cereal-based foods, which are deficient in lysine and high in methionine [39]. In addition, branched-chained amino acids have beneficial effects on the human immune function, protein synthesis, and gut health [40]. The lentil protein cysteine content, especially, was notably higher than the proposed dietary requirements (amino acid score >226%), which is indicate better nutritional values than other pulses that contain minimal levels of this amino acid [36]. Cysteine serves as a substrate for the biosynthesis of glutathione, which is a powerful antioxidant that protects the body against oxidative damage, and has been known to promote fertility in males [41,42]. Valine content of lentil proteins was also found to be significantly higher (5.1–5.4%) when compared with soybean (4.7%), peas (4.5%), or chickpea (4.0%) [43,44,45]. The isolated lentil proteins all had slightly higher amino acids than previously reported literature data [44,46,47]. Two notable differences with previous literature reports on lentil seed proteins are the serine and glycine. In the present work, serine contents of the isolated proteins are higher (>5.1%) than previously documented results (∼4.7%), but glycine levels in ISO (3.4%), and MEM_NaOH (3.8%) are lower (4.1%).

Figure 1 shows that all the protein isolates contain the typical bands consistent with legumin and vicilin-like proteins. Legumins are hexamers made up of acidic (∼40 kDa) and basic (∼19 kDa) subunits linked together by a disulfide bond. MEM_NaOH were primarily made up of MW bands of <47 kDa, indicating that legumins are the major polypeptides present [5,48,49]. Similarly, the isolates had similar recurring bands at ∼15 kDa, ∼19 kDa, and ∼23 kDa, corresponding to the β-subunit of legumin. Vicilin is a trimer consisting of α, β, and γ-polypeptide subunits with molecular weights between 50 kDa and 67 kDa and can be found in MEM_NaCl and ISO [5,50]. Subunits of 70 kDa and 90 kDa present in ISO on the other hand, correspond to convicilins. These results are similar to those reported in literature for other isolated lentil proteins, which also had corresponding bands between 14 kDa and 75 kDa [15,51,52]. However, absence of the convicilins from the membrane isolates under reducing condition (Figure 1A) indicates the proteins were held together by disulfide bonds. When β-mercaptoethanol was added, there was a change in the major polypeptide bands of all isolates including the breakdown of β-legumin subunits to yield lower MW (<14 kDa) polypeptides. This indicates that the legumins are also stabilised by disulfide bonds, which is in contrast to a previously report, which suggested only subtle differences in the protein bands under reduced and non-reduced conditions [53]. However, the absence of significant changes in the band intensity of vicilins in ISO and MEM_NaCl under reducing condition suggests that the 55–65 kDa polypeptides lack disulfide bonds.

Aromatic amino acids like Trp, Tyr, and Phe can emit fluorescence spectra with maximum values at 350, 303, and 280 nm, respectively when they are excited in the UV region [54]. These emission wavelengths reflect conformational changes, which are dependent on exposure of the aromatic amino acids to the hydrophilic environment [55]. Since this gives more information about how the proteins are folded, it can also be used to predict other functional properties of the protein such as solubility, which involves protein–solvent interactions [14]. The results in Figure 2 indicate that the indole group in the Trp residues of MEM_NaCl at pH 9 were positioned in a more polar environment, while the Trp residues of ISO, and MEM_NaOH were in a more hydrophobic environment. In general, MEM_NaCl was the only sample that had a fluorescence peak at each pH, and except for pH 9, its FI also remained comparatively the highest. This could be attributed to the higher content of Trp and Tyr residues in MEM_NaCl but also indicates a more folded conformation when compared to ISO and MEM_NaOH. MEM_NaOH on the other hand, only had a peak at pH 9, signifying a very loose structural conformation at pH 3, 5, or 7, which is similar to ISO at pH 3 and 5. In both cases, the lower FI indicate a more extensive interactions of ISO and MEM_NaOH with the hydrophilic environment when compared to MEM_NaCl [55]. The λmax results obtained in this work are generally comparable to the 322–332 nm, and 333 nm reported for lentil protein isolate and Bambara proteins, respectively [31,35].

The results also show that β-sheet structures of MEM_NaCl were greatly reduced in comparison with the other samples (Table 3). However, all the protein samples had more β-sheet than helical conformations. The presence of high ratios of random coils indicates highly disordered and less compact protein structures at pH 7 and 9. Loss of the compact nature of the protein structures could be associated with a higher protein solubility as shown by the improved solubility values reported at pH 7 and 9.

The effect of pH on the tertiary structures of ISO, MEM_NaOH, and MEM_NaCl, which are dependent on the location of aromatic amino acid residues (Phe, Tyr, and Trp), was investigated using near-UV (250–320 nm) CD as shown in Figure 3. The CD signal of each protein depends on the number and proximity of the aromatic amino acid residues, degree of H-bonding, polar groups present, disulfide bonds, and nature of the chromophores [56]. At pH 3, the lentil proteins had a positive peak ellipticity at 255–261 nm, which is consistent with Phe residues within a hydrophobic atmosphere. Another peak indicating the presence of Trp can also be seen at 290–305 nm for ISO and MEM_NaCl. Similar Phe and Trp peaks were also found at pH 5 but transitioned into more intense Phe peaks at pH 7 and 9 for all samples, indicating a degree of denaturation had occurred at that pH. More intense positive peaks and lack of distinct Trp transition at pH 7 and 9 also suggest that a shift into a more hydrophilic exterior had occurred in the location of aromatic residues within the protein conformation. However, the MEM_NaOH protein had the least tertiary structure as evident in the almost zero CD values at all the pH values, which suggests substantial protein denaturation during alkaline extraction. It is also interesting to note that all the proteins show extensive denaturation at pH 5, which is close to the isoelectric point and indicate substantial structural disorganization. In contrast, the ISO and MEM-NaCl proteins regained more compact structural conformations at pH 7 and 9 than at pH 3 and 5. The results suggest that for ISO and MEM-NaCl proteins, compact structures were formed at pH 7 and 9, probably as a result of increased net charges that resulted in translocation of the aromatic amino acids away from the hydrophilic surface and into the hydrophobic core.

Figure 4 indicates protein–protein interactions occurred, which is consistent with the isolation protocols that involve aggregation. However, the smoother appearance of the MEM_NaOH and MEM_NaCl when compared to ISO indicate that membrane isolation has less negative impact on the protein structural properties than the isoelectric pH precipitation. The MEM_NaCl had the highest enthalpy of denaturation (Table 4), which is consistent with a more folded conformation and confirms that this protein underwent less structural disorganization when compared to the ISO and MEM_NaOH. The low yield of MEM_NaCl (Table 5) could be attributed to solubilization of only the albumins and globulins while the glutelins were excluded. In contrast, NaOH is able to solubilize most of the protein categories (except prolamins), hence higher yields of MEM_NaOH and ISO. Protein quality not only depends on the pattern and number of amino acids but also on the amount that will be made available for absorption after digestion. Protein digestibility is, therefore, also an important parameter when evaluating the nutritive value of protein food products. The presence of antinutritional compounds like phytic acid, and tannins, as well as the large size and folded conformation of plant storage proteins, lower the digestibility of raw lentil flours [57]. To improve protein digestibility and nutritional quality, the effects on these antinutrients can be reduced by various processing methods including the extraction and fractionation. The results obtained for ISO (Table 5) is higher than the 83.2% and 85.4% previously reported values for isoelectric pH precipitated lentil protein isolates [33,51]. Although the membrane isolates had significantly lower digestibility in comparison, they were comparable or even higher then that of quinoa protein isolate (78.4%) and other plant protein products such as flaxseed (68.0%), barley (77.5%), chickpea (77.0%), and common bean (73.5%) [58,59,60]. ISO on the other hand, performed admirably when compared with animal protein products that are usually known to have higher digestibility. According to Carbonaro and others [58], chicken meat was slightly more digestible (92.0%) whereas ISO had a higher digestibility than mozzarella cheese (87.0%) and pasteurized milk (84.0%). The lower digestibility of the membrane isolated proteins indicates presence in a more native conformation (folded) when compared to the ISO, which may have been denatured (greater unfolding) during acid precipitation.

Surface hydrophobicity (S_o_) is an important means of characterizing proteins and evaluating their surface related functional properties. It reflects the change in protein conformation resulting from the extent of intermolecular protein interactions [61]. Variations in the S_o_ of the different proteins (Table 5) could be attributed to their varied amino acid compositions, resulting in a higher or lower exposure of aromatic and aliphatic amino acid residues [62]. Denaturation caused by isoelectric precipitation increases the exposure of hydrophobic groups of ISO, which could be responsible for the higher S_o_ when compared to the milder membrane filtration process used to produce MEM_NaOH [63]. The possible influence of the degree of denaturation (caused by pH) on hydrophobicity is in agreement with the result of Arogundadeetal. Ref. [61], who showed that the S_o_ of their African yam bean protein isolate obtained from ultrafiltration was lower than that of isoelectric precipitation. Even though the MEM_NaCl was also a membrane isolated product, the higher S_o_ could be due to the higher contents of Tyr and Trp (Table 2). Alonso and others [15] concluded that the method of extraction had no major impact on their S_o_ but noted, however, that there was a significant difference between the hydrophobicity of the membrane and isoelectric pH precipitated lentil protein isolates.

Solubility is primarily dependent on the equilibrium between protein–solvent and protein–protein interactions, which influence other functional properties [2]. The solubility profile at various pH levels can, therefore, serve as a useful indicator of the protein’s efficiency in food systems and the degree of protein denaturation induced by the extraction process [50]. Solubility profile obtained in Figure 5 is consistent with the solubility of most pulse proteins, with higher values at alkaline pH when compared to the acidic region [5,64]. The significant decreases in the solubility of the lentil proteins at pH 4 and pH 5 (<28%), which represent the isoelectric points of pulse proteins are similar to the low solubility values reported in literature. At the isoelectric point, there is a reduction in the electrostatic repulsion due to weakly charged proteins, which leads to aggregation of proteins and hence reduced solubility [24]. The isoelectric point of the proteins also reveal that their major proteins are acidic in nature, which is evident from high amount of acidic amino acids (glutamic and aspartic) present in their amino acid composition. The higher solubility of ISO compared with MEM_NaOH at pH ranges above its isoelectric point is consistent with previous findings for pea and lentil proteins [24,65]. Also similar to their findings are the characteristically higher solubility at neutral pH for concentrates obtained though ultrafiltration when compared to isoelectric precipitation. The difference in solubility is usually attributed to an inverse correlation with their surface hydrophobicity [24,66], but that was not the case with the present study. For instance, MEM_NaOH had a significantly low surface hydrophobicity compared to ISO and MEM_NaCl but was still not the most soluble of the three samples at any pH recorded. This emphasizes the degree of influence of other surface and structural characteristics on protein solubility, especially conformation and level of protein–protein interactions.

Coagulation is defined as a reversible change in the structure of proteins, which is commonly induced by heat, but can also be brought about by acids, mechanical action, or addition of coagulants [10]. Therefore, HC is an index used to estimate the susceptibility of proteins to heating and is characterised by substantial reductions in solubility resulting from aggregation of unfolded protein molecules [10]. It has been suggested that protein thermal stability has a positive correlation with the presence of hydrophobic amino acids [67]. This may be responsible for the higher HC of ISO, which has lower contents of Tyr and Trp, when compared to the more heat-stable MEM_NaCl (Figure 6).

The ability of proteins to hold water or oil is another important functional property when formulating certain foods because it affects the texture, flavor retention, mouthfeel, and shelf life of the food product [49]. Inability of a protein to bind water could result in stiff and dry food products. The significantly higher WHC of MEM_NaOH (Table 5) was reported for lentil protein isolates prepared using the freeze-drying method [32]. The WHC values in the current study are however lower than the 2.2–4.2 mL/g (or g/g, assuming that 1 mL of water is equivalent to 1 g of water) reported for other lentil protein isolates [15,30,65,68]. In terms of OHC, higher surface hydrophobicity has been reported to be one of the prerequisites for more favourable results [23]. However, that was not the case with ISO, which had a lower surface hydrophobicity than MEM_NaOH but exhibited better OHC. The results suggest that other parameters including the protein exposed surface area and charge may have contributed to the OHC of the proteins.

Emulsions are generally formed when immiscible liquids are mixed to produce a continuous phase with one liquid dispersed as small droplets in the other. Due to the high surface tension that prevents mixing of the two immiscible liquids and the high level of thermodynamical instability of the emulsified product, emulsifiers such as lentil proteins can be used to enhance formation and stability of emulsions [49]. During the emulsification process, proteins migrate to the oil–water interface where they are adsorbed and form viscoelastic films around the oil droplets such that the hydrophobic and hydrophilic groups of the protein are positioned towards the oil and aqueous phases, respectively [11,49]. The surface area and droplet size of the oil in the emulsions formed using MEM_NaCl protein remained relatively unchanged across various concentrations and pH range, suggesting minimal effect of pH on protein unfolding. ISO and MEM_NaOH proteins formed emulsions with bigger oil droplet sizes at pH 3 and 5 when compared to emulsions formed by MEM_NaCl protein (Figure 7). The oil droplet size of ISO, however, doubled for all concentrations when the pH of the emulsion was increased from pH 3 to pH 5 but was drastically reduced at pH 7 and pH 9. This is a clear indication that at pH 5, which is close to the isoelectric point, there was limited net neutral charge on the protein’s surface and therefore, enhanced oil droplet aggregation due to weak electrostatic repulsions. As a result, most emulsions had the lowest oil droplet size at pH 9 where higher net charge is maintained. Overall, pH change had more effect on emulsion formation than protein concentration, which indicates the strong influence of the environment. The results are comparable to previous reports for pea and isolated lentil proteins [5,69,70].

Changes in the oil droplet size of the emulsions were measured after a 30-min interval to estimate stability (ES). As seen in Figure 8, ES varied with changes in pH and protein concentration. At 10 mg/mL for example, ISO demonstrated the highest emulsion stability at pH 7 and pH 9 (75% and 117%), while the reverse was the case for pH 3 and pH 5 using the same concentration (41% and 83%). This is consistent with the bigger oil droplet sizes at acidic pH and increased solubility at pH 7 and pH 9 for ISO, which emphasizes the inverse relationship between oil droplet size and emulsion stability. Similarly, MEM_NaCl protein had lower solubility and formed emulsions with bigger oil droplets and least stability at pH 7 and 9 when compared to the other isolated proteins.

FC and FS are generally dependent on the interfacial film formed by proteins, which incorporate air bubbles in suspension and reduce the rate of coalescence [71]. In agreement with the present results (Figure 9), Boye and others [65] reported better foaming properties for isolates prepared by isoelectric precipitation (79%) when compared to those prepared by ultrafiltration (69%) at pH 7. A similar trend was reported by for yam bean protein isolates [61] but the opposite was observed for lentil protein [15] and hemp seed proteins [14], respectively. Since, solubility is one of the prerequisites for foam formation, the differences in FC may be attributed to variations in the solubility of the proteins. For example, Alonso and others [15] reported that the lentil proteins prepared by ultrafiltration had higher solubility across all pH range when compared with the isoelectric precipitated protein. However, ISO had significantly higher solubility than MEM_NaOH at pH > 5.0, which could account for the higher FC of ISO in this study. The higher FC in ISO may result from its enhanced ability to form air bubbles a result of its greater unfolded conformation, which could be inferred from its higher interaction and solubility in water. The FC for MEM_NaOH and MEM_NaCl was observed to increase from pH 3 to pH 5 then reduced to attain the least at pH 9, which is the point of highest solubility but suggest a rigid structure with limited ability to unfold and encapsulate air bubbles. In contrast, the higher FC of ISO at neutral to alkaline pH values may be due to structural properties including the flexibility of the lentil proteins. Higher stability (FS) at acidic pH values could result from their ability to form cohesive viscous membranes that could maintain a stable foam by reducing foam drainage (Figure 10) [14]. Specifically, lower solubility observed at the acidic pH ranges compared with the alkaline pH ranges, increases protein–protein interaction responsible in the formation of the interfacial membrane. Nonetheless, the lowest FS was 73% while the highest FS was 100%, indicating that foams produced by the lentil proteins were generally very stable. FS in this work at pH 7 (75–100%) is significantly higher than the 6–45% [15], and <44% [63] previously reported values, but similar to the 84% average reported by Jarpa-Parra and others [72].

## 5. Conclusions

This work has demonstrated the significant role of protein isolation method in defining structure and function. Crude protein content, surface hydrophobicity, and functional properties of ISO, MEM_NaOH, and MEM_NaCl, were significantly different. Where applicable, the effects of pH changes on the various physicochemical and functional properties of the lentil proteins were apparent, which emphasizes the impact of the protein environment on structural conformation, as well as functionality. For example, higher solubility was observed at alkaline pH values, while higher FS and WHC were achieved at acidic pH values. All isolates had polypeptide bands that are consistent with legumin and vicilin-like proteins but MEM_NaOH was primarily made up of legumins in contrast to MEM_NaCl, which had a higher ratio of the vicilins. Overall, the isolated lentil seed proteins demonstrated excellent functional properties depending on the environment pH, which provides protein ingredient choices that can be used for a variety of food product formulations.

## Figures and Tables

**Table 1:** Proximate composition of defatted lentil four (DEF), isoelectric pH-precipitated isolate (ISO), and membrane isolates from alkaline (MEM_NaOH) and salt (MEM_NaCl) solution extracts.

	DEF	ISO	MEM_NaOH	MEM_NaCl
Moisture content (%)	7.12 ± 0.54 ^a^	5.24 ± 0.72 ^b^	8.59 ± 0.52 ^a^	2.64 ± 0.83 ^c^
Crude protein (%)	29.35 ± 0.45 ^d^	86.13 ± 1.02 ^b^	82.55 ± 0.54 ^c^	90.28 ± 0.67 ^a^
Crude fibre (%)	4.41 ± 0.25 ^a^	0.10 ± 0.07 ^b^	0.02 ± 0.02 ^c^	0.02 ± 0.01 ^c^
Fat (%)	1.36 ± 0.66 ^b^	0.35 ± 0.13 ^d^	1.57 ± 0.23 ^a^	0.39 ± 0.15 ^c^
Ash (%)	2.96 ± 0.04 ^d^	6.00 ± 0.21 ^a^	3.72 ± 0.17 ^c^	4.75 ± 0.05 ^b^
Non-Fibre Carbohydrates (%)	60.99 ± 1.32 ^a^	6.48 ± 1.43 ^c^	11.24 ± 0.95 ^b^	3.61 ± 0.76 ^c^

Each value is the mean and standard deviation of triplicate determinations. Within each row, different letters (^a^, ^b^, ^c^, and ^d^) indicate significant differences at *p* < 0.05.

**Table 2:** Percentage amino acid composition of lentil seed protein isolates *.

AA	ISO	MEM_NaOH	MEM_NaCl	Amino Acid Score (%)
Asx	12.29 ± 0.17	12.89 ± 0.12	11.79 ± 0.38	
Thr ^1^	3.78 ± 0.00	4.06 ± 0.02	4.76 ± 0.06	118 ^a^, 108 ^b^, 140 ^c^
Ser	5.40 ± 0.08	5.38 ± 0.14	5.12 ± 0.09	
Glx	18.01 ± 0.19	17.71 ± 0.09	15.77 ± 0.11	
Pro	4.19 ± 0.06	4.69 ± 0.00	4.21 ± 0.04	
Gly	3.47 ± 0.13	3.79 ± 0.04	4.02 ± 0.07	
Ala	3.84 ± 0.06	3.85 ± 0.09	4.84 ± 0.19	
Cys ^1^	0.96 ± 0.56	1.41 ± 0.13	1.38 ± 0.13	163 ^a^, 153 ^b^, 164 ^c^
Val ^1^	5.21 ± 0.10	5.33 ± 0.01	5.49 ± 0.05	95 ^a^, 83 ^b^, 94 ^c^
Met ^1^	1.01 ± 0.04	1.31 ± 0.06	1.33 ± 0.02	47 ^a^, 52 ^b^, 56 ^c^
Ile ^1^	4.52 ± 0.42	4.78 ± 0.49	4.26 ± 0.41	115 ^a^, 104 ^b^, 102 ^c^
Leu ^1^	8.43 ± 0.04	8.18 ± 0.09	7.31 ± 0.05	103 ^a^, 85 ^b^, 83 ^c^
Tyr	3.55 ± 0.02	3.56 ± 0.16	4.18 ± 0.40	
Phe	5.81 ± 0.06	5.74 ± 0.02	5.08 ± 0.02	
His ^1^	2.32 ± 0.25	2.41 ± 0.35	2.36 ± 0.21	120 ^a^, 108 ^b^, 112 ^c^
Lys ^1^	6.75 ± 0.18	6.54 ± 0.13	8.78 ± 0.39	110 ^a^, 90 ^b^, 135 ^c^
Arg	8.94 ± 0.25	6.92 ± 0.11	6.58 ± 0.06	
Trp ^1^	0.85 ± 0.06	1.01 ± 0.05	1.20 ± 0.03	107 ^a^, 106 ^b^, 136 ^c^
AAA	10.21 ± 0.01	10.31 ± 0.10	10.46 ± 0.40	
BCAA	18.16 ± 0.37	18.30 ± 0.60	17.06 ± 0.41	
HAA	38.38 ± 0.95	39.87 ± 0.78	39.28 ± 0.32	
PCAA	18.01 ± 0.68	15.87 ± 0.59	17.72 ± 0.54	
NCAA	30.30 ± 0.02	30.61 ± 0.21	27.56 ± 0.49	
SCAA	1.97 ± 0.60	2.72 ± 0.19	2.70 ± 0.15	

* ISO (isoelectric pH precipitation); membrane isolated proteins from alkaline (MEM_NaOH) and salt (MEM_NaCl) extracts; Asx = aspartic acid + asparagine; Glx = glutamic acid + glutamine; AAA = aromatic amino acids; BCAA = branched-chain amino acids; HAA = hydrophobic amino acids; NCAA = negatively charged amino acids; PCAA = positively charged amino acids; SCAA = sulfur-containing amino acids; ^1^ Essential amino acids; amino acid score calculated with EAA requirements for adults according to WHO and FAO (2007).^a^ ISO, ^b^ MEM_NaOH, ^c^ MEM_NaCl.

**Table 3:** Secondary structure fractions at different pH values of isoelectric pH precipitated isolate (ISO), and membrane isolated proteins from alkaline (MEM_NaOH) and salt (MEM_NaCl) solution extracts.

**ISO**				
pH	3	5	7	9
α-helix	2.37 ± 0.03	2.52 ± 0.03	7.65 ± 0.28	6.05 ± 0.28
β-sheet	17.60 ± 0.07	17.00 ± 0.14	9.75 ± 0.64	12.38 ± 0.39
Turns	18.90 ± 0.00	18.85 ± 0.21	14.20 ± 0.28	13.65 ± 0.64
Unordered	41.25 ± 0.07	42.10 ± 0.14	50.95 ± 1.48	49.55 ± 1.34
Total	100.10 ± 0.00	100.00 ± 0.00	99.95 ± 0.07	100.05 ± 0.07
	100.10 ± 0.28	100.00 ± 0.07	99.95 ± 3.61	100.05 ± 3.32
**MEM_NaOH**				
pH	3	5	7	9
α-helix	2.45 ± 0.00	3.35 ± 0.14	3.92 ± 0.25	5.65 ± 0.64
β-sheet	17.57 ± 0.03	14.87 ± 0.39	13.55 ± 0.64	12.00 ± 0.21
Turns	18.50 ± 0.00	17.80 ± 0.28	16.80 ± 1.27	14.40 ± 0.28
Unordered	41.50 ± 0.00	45.75 ± 1.20	48.25 ± 2.19	50.30 ± 0.14
Total	100.05 ± 0.07	100.00 ± 0.14	100.00 ± 0.14	100.00 ± 0.00
	100.05 ± 0.07	100.00 ± 2.54	100.00 ± 5.23	100.00 ± 2.12
**MEM_NaCl**				
pH	3	5	7	9
α-helix	6.20 ± 0.28	7.78 ± 0.60	5.08 ± 0.25	6.13 ± 0.32
β-sheet	11.85 ± 0.85	13.25 ± 0.28	13.17 ± 0.39	12.20 ± 0.49
Turns	17.40 ± 0.42	17.95 ± 0.35	13.35 ± 0.92	12.55 ± 0.49
Unordered	46.50 ± 0.71	40.00 ± 2.12	50.20 ± 1.27	50.75 ± 0.21
Total	100 ± 0.00	100.00 ± 0.00	100.05 ± 0.07	99.95 ± 0.07
	100 ± 3.39	100.00 ± 4.24	100.05 ± 3.46	99.95 ± 2.33

**Table 4:** Thermal properties of isoelectric pH precipitated isolate (ISO), and membrane isolated proteins from alkaline (MEM_NaOH) and salt (MEM_NaCl) solution extracts obtained using differential scanning calorimetry *.

Sample	Onset T_o_ (°C)	Maximum T_p_ (°C)	Area ∆H (J/g of Sample)
ISO	86.43 ± 0.49 ^a^	90.66 ± 0.10	0.16 ± 0.09 ^b^
MEM_NaOH	86.19 ± 0.13 ^b^	90.72 ± 0.12	0.19 ± 0.04 ^b^
MEM_NaCl	85.86 ± 0.34 ^c^	90.90 ± 0.06 ^a^	0.34 ± 0.00 ^a^

* T_o_, onset of denaturation temperature; T_p_, peak temperature of denaturation. For each column, mean values with different letters are significantly different (*p* < 0.05).

**Table 5:** Yield, digestibility, least gelation concentration (LGC), surface hydrophobicity, water holding capacity (WHC, pH 7.0), and oil holding capacity (OHC) of isoelectric precipitated protein isolate (ISO), and membrane protein isolates from alkaline (MEM_NaOH) and salt (MEM_NaCl) solution extracts.

	ISO	MEM_NaOH	MEM_NaCl
Protein yield	48.45 ± 0.76 ^a^	35.05 ± 1.64 ^b^	13.35 ± 0.05 ^c^
Protein digestibility	89.82 ± 0.13 ^a^	77.97 ± 0.5 ^b^	77.61 ± 0.00 ^b^
LGC	11.50 ± 0.71 ^a^	11 ± 0.00 ^a^	10.75 ± 0.35 ^a^
Surface hydrophobicity	5.14 ± 0.11 ^b^	1.06 ± 0.02 ^c^	5.80 ± 0.01 ^a^
WHC (40 mg/mL)	0.15 ± 0.05 ^b^	0.47 ± 0.67 ^a^	0.10 ± 0.08 ^b^
OHC (40 mg/mL)	1.63 ± 1.97 ^a^	1.07 ± 1.48 ^b^	0.92 ± 1.21 ^b^

Each value is the mean and standard deviation of triplicate determinations. Different superscript characters (^a^, ^b^, and ^c^) indicate significant differences at *p* < 0.05 level within a row.

## Data Availability

Data are available from the corresponding author.
